# Progress in Disease of Digestive Organs

**Published:** 1903-11-28

**Authors:** 


### PROGRESS IN DISEASE OF DIGESTIVE ORGANS.

(*Continued from page* 132.)

**Dilatation of the Stomach.**—Since Fagge described two cases of acute dilatation in 1873, Thompson has collected ten cases and Mayo Robson, and Moynihan have recorded six others.[11] The symptoms are sudden vomiting, diminution of urine, and the physical signs of dilatation. The theories as to its production are that the duodenum is kinked by the descent of the stomach or compressed by the veins at the root of the mesentery, or that its third part is pressed upon by the already full stomach. Box and Battle[12] have recently published a case which recovered, in which the semi-prone position was found useful. They considered that pressure on the duodenum by the mesenteric veins was not responsible in this case, as diarrhœa, which would have left that organ empty, did not occur till after the acute dilatation. This case supported the supposed toxic origin of the disease, as suppuration had occurred in an ovariotomy wound, but in a case of Stewart's[13] which was fatal the ischio-rectal abscess for which the patient was admitted was practically healed when the symptoms set in. In this case the bladder, the beginning of the duodenum, and the right ventricle were also dilated, and the kidneys were granular and cystic. Obscure changes in the sympathetic were suggested as a cause, or the "cryptogenic septicæmia" of Leube. The commoner condition of atonic dilatation has been treated successfully in one instance by Pierce[14] according to Hendall's method. This consists of continuous heat to the abdomen, *absolute* rest in bed, and ordinary liberal diet regardless of the pain it causes. No drugs or stimulants are given. In three months the patient who had been ill for 20 years was practically cured.

**Gastric Crises.**—These are always associated with early tabes, and in nearly 18 per cent. syphilis was found by Ewald.[15] In his series the intervals averaged 2 years and 10 months, but in late stages the attacks may occur every day. Subacidity is usual and may be extreme. Constipation is common. Intestinal and anal crises are much rarer. The diagnosis is occasionally difficult, and operation for gastric ulcer and ovariotomy have been performed in these cases. No method of treatment will cut short the attacks, but general treatment may be combined with morphia hypodermically or cocaine injected into the spinal canal.

**Abdominal Pain**, when spontaneous and acute, is often a sign of urgent need for surgical treatment. Important co-existing symptoms are, says Lynn Thomas,[16] the rigid abdomen, the peculiar facies, and the small rapid pulse. Liver dulness is unimportant, pyrexia variable, and the vomiting less significant than other symptoms; it is continuous and severe in acute obstruction and in gangrenous appendicitis is intermittent, and associated with hiccough or occasional diaphragmatic spasm. The exact localisation of the pain at its onset may give the clue to the diagnosis. Extreme rigidity of the abdomen suggests perforation. He quotes cases to show how thrombosis of the superior mesenteric vein may simulate obstruction of the bowel, and others of tubal gestation and perforated gastric ulcer which might probably have recovered without operation. Hilton was the first to study "referred" pain, and recently Head has investigated it elaborately. Mansell Moullin has recently shown that though the viscera are not sensitive, the abdominal walls are acutely so, and Bishop draws an analogy between the abdominal rigidity in early peritonitis and that seen in the muscles about an inflamed joint which was shown by Hilton to depend on the anatomical arrangement of the nerve supply. As showing the danger of error in interpreting referred pain, a case has been quoted in which ovariotomy was performed, and it was afterwards found that the pain was due to impacted calculus low down in the ureter.[17] Bottentuit mentions cases of mucous colitis, which have been diagnosed as hepatic or renal colic.

**Muco-membranous Colitis** appears as a chronic disease not due to any particular organism but in which the stools contain many bacteria of the colon type. Bottentuit[26] classifies the cases according as they suffer from diarrhœa, constipation, or a combination of the two. The last two are about equally common, the first rare. Dyspepsia, gastric dilatation, enteroptosis, and hyperchlorhydria are common accompaniments. Real appendicitis is rare. The diagnosis rests on the appearance of the stools. It is rarely fatal, but very obstinate. Milk diet and violent purges are useless, bland diet and gentle laxatives, or bismuth and antiseptics, according to the indications, with lavage and, if possible, a sojourn at Plombières, are advised.

**Nutrition.**—The condition known as sitophobia has been ascribed to psychical disease, but it is considered by Einhorn[18] to be compatible with perfect rationality, although it must be combated by setting aside the premisses on which the patient argues. The fear of pain or discomfort leads him to starve himself until nutrition seriously suffers. The treatment is feeding; at first easily digestible liquid food gradually increased as nutrition improves. Gastric sedatives may be employed at first. On the other hand a class of cases is described (hypersthenic dyspepsia) in which actual incapacity to use any excess of food-stuff exists. Bardet[19] considers that the normal individual can absorb several times the amount of food absolutely required, but that in middle life the effect of this is seen in undue irritability of the digestive system. Careful, restricted diet and alkalies are indicated. The influence of treatment on body weight, when either excessive or deficient, has been reviewed by Einhorn.[20] In the former case he recommends regulated exercise, stopping short of fatigue and exhaustion, and a proteid diet, either, skim-milk or meat, if weight is actually increasing, or exercises on a mixed diet if weight is merely stationary. In the latter, exercise in the open air is also necessary to improve appetite, and in the increased dietary milk and butter should take a prominent place. These observations are in consonance with the views of Lütje recently confirmed by Kaufman and Mohr,[21] who consider that an index of the amount of retained nitrogen in forced feeding can be found in the amount of inorganic salts required for the formation of protoplasm. Krug, however, thinks that the nitrogen is retained not as protoplasm but as a reserve albumin analogously to the storage of glycogen. The inorganic salts are undoubtedly of importance to nutrition, as is shown in the case of milk, in which in various animals the amount of lime salts present is directly proportional to the rate of growth of the suckling.[22] With regard to sodium chloride, some new facts have been disclosed which seem to throw doubts on Bunge's well-known theory. Whether the excess of this salt usually taken throws any extra strain on the kidneys has not yet been decided; it apparently protects the kidneys from the action of strong urea solutions. In considering the general therapeutics of nutrition, Robin and Binet[23] consider that sea air and bathing are important where metabolism has to be stimulated, as in rachitis, scrofula, gout, auto-intoxication, hyposthenic dyspepsia, neurasthenia, and convalescence from fevers; in anæmia and diabetes only when oxidation is deficient and in tuberculosis of bones and glands where repair is in progress. In phthisis they are contra-indicated. Of special food stuffs Plasmon may be used in all cases where milk is indicated as a tissue builder. It is less likely to cause diarrhœa though equally inclined to constipate, and its poorness in fat and carbohydrates renders it less liable to set up gastric disturbance (Douglas).[24]

**Tuberculous Peritonitis.**—The question of laparotomy in this condition has been discussed by Sutherland[49] and Dyce Duckworth.[50] The latter recommends laparotomy in cases which are severe, but the former quotes 27 cases medically and 14 surgically in which the last were not appreciably affected by the treatment. They were, however, more severe. Shattock among older patients had a mortality of 13·2 per cent. in 52 surgical cases and 46 medical. Tuberculous pleurisy does not materially affect the prognosis, *cæteris paribus*, but it is inadvisable to operate in acute cases, as fluid usually reaccumulates (Guthrie), and these acutely beginning cases often do well if succeeded by organising changes (Sutherland).

[11] Lancet, 1903, i., p. 1646. [12] Ibid., p. 1031. [13] Ibid., p. 1303. [14] Brit. Med. Jour., 1901, i., p. 1150. [15] Med. Rec., 1903, ii., p. 113. [16] Brit. Med. Jour., 1903, ii., p. 186. [17] Lancet, 1903, ii., p. 766. [26] Brit. Med. Jour., 1903, i., p. 1488. [18] Amer. Jour. Med. Sci., Aug., 1903. [19] Med. Rec., 1903, ii., p. 268. [20] Med. Rec., 1903, ii., p. 91. [21] Ibid., 1903, i., p. 471. [22] Brit. Med. Jour., 1903, ii., p. 93. [23] Med. Rec., 1903, ii., p. 268. [24] Scottish Med. and Surg. Jour., March, 1903. [49] Med. Chi., July, 1903. [50] Clin. Jour., 1903, p. 209.

*To be continued.*

